# High-Intensity Interval Training Improves Cardiovascular Fitness and Induces Left-Ventricular Hypertrophy During Off-Season

**DOI:** 10.3390/jfmk10030271

**Published:** 2025-07-16

**Authors:** Tomas Venckunas, Birute Gumauskiene, Pornpimol Muanjai, Joan Aureli Cadefau, Sigitas Kamandulis

**Affiliations:** 1 Institute of Sport Science and Innovations, Lithuanian Sports University, Sporto Str. 6, 44221 Kaunas, Lithuania; pornpimolm@buu.ac.th (P.M.); sigitas.kamandulis@lsu.lt (S.K.); 2 Department of Cardiology, Medical Academy, Lithuanian University of Health Sciences, 44307 Kaunas, Lithuania; 3 Institut Nacional d’Educació Física de Catalunya (INEFC), Universitat de Barcelona (UB), 08038 Barcelona, Spain; jcadefau@gencat.cat

**Keywords:** high-intensity interval training, aerobic capacity, athlete’s heart, cardiac hypertrophy

## Abstract

**Background**: Well-designed endurance training leads to improved cardiovascular fitness and sports performance in prolonged exercise tasks, with the adaptations depending on multiple factors, including the training modality and the population in question. It is still disputable how the type of training affects myocardial remodeling, and the information on myocardial remodeling by high-intensity interval training (HIIT) is particularly scarce. **Methods**: The current study investigated changes in cardiac structure after volume-progressive HIIT in running mode. As part of their conditioning program, amateur athletes (mean ± SD age of 18.2 ± 1.0 years) exclusively conducted HIIT in a volume-progressive fashion over 7 weeks (a total of 21 sessions). Peak oxygen uptake as well as 200 m and 2000 m running performance were measured, and transthoracic two-dimensional echocardiography was conducted before and after the intervention. **Results**: Training improved running performance, increased the peak oxygen uptake and left atrium diameter (from 32.0 ± 2.5 to 33.5 ± 2.3 mm; *p* = 0.01), and induced ~11% thickening of the left-ventricular posterior wall (7.5 ± 0.7 to 8.2 ± 0.4 mm; *p* = 0.01) and interventricular septum (7.6 ± 0.7 to 8.6 ± 0.9 mm; *p* = 0.02), but not the dilation of left-ventricular, right-ventricular, or right atrium chambers. **Conclusions**: HIIT of just 127 km of running per 8.5 h during 7 weeks was sufficient to improve aerobic capacity and running performance, and induce left-ventricular wall hypertrophy and left atrium dilation, in young healthy athletes.

## 1. Introduction

Regular intense endurance exercise training leads to cardiac adaptation, termed “athlete’s heart”, which typically encompasses an increase in ventricular wall thickness and chamber volume along with improved cardiac function [1,2,3,4]. It remains elusive how different types of endurance training affect cardiac remodeling as the literature is abundant with discrepant findings, and large heterogeneity in cardiac adaptation is reported between individuals [5]. For instance, in top-level swimmers training > 3 h per day, left-ventricular (LV) wall thickness increased with no evidence of chamber dilation, i.e., concentric hypertrophy occurred [6], while 3 months of intensified training in college swimmers was reported to induce eccentric cardiac remodeling [7], similarly to the augmentation of training volume in well-trained rowers [3]. Others have reported that intensified high-volume training in army recruits [8] and professional cyclists [9] leads to balanced dilation and wall thickening of the LV. A high training volume elicited LV wall thickening but not further dilation in experienced endurance runners [10,11,12], while in professional road cyclists, an increase in LV volume and LV wall thinning was reported during the progress of their career [13]. On the other end of the dynamic sport spectrum, sprint training composed of repetitive short-activity bursts of (close to) maximal intensity with a full recovery between the efforts does not seem to result in any significant cardiac remodeling, even in high-level athletes practicing it on regular basis [14,15,16,17,18]. However, a recent study implementing sprint training with short (incomplete) recovery between efforts effectively showed a dilation of the LV chamber [19].

High-intensity interval training (HIIT) is an effective and time-efficient means of increasing aerobic capacity [20,21,22,23], and its popularity necessitates a better understanding of the various (mal)adaptations it is capable of inducing. Although high-intensity interval cycling training performed at two different intensities every session until exhaustion for 6 weeks did not change the LV structure of well-trained endurance athletes [23], it has recently been proposed that the initiation of endurance training early in life may lead to a more eccentric type of LV hypertrophy [24]. In agreement, endurance training in adolescent skiers clearly led to LV chamber enlargement during a period of 3 years [25]. However, cardiac adaptations to only HIIT at a young age could substantially differ from those of predominantly continuous training sessions. The current longitudinal study was instigated by our earlier observation that middle-distance runners (who employ HIIT as a major component in their development) have the same chamber volume but smaller LV wall thickness, i.e., a somewhat more eccentric LV and lower LV mass in comparison to marathon runners [10], a finding that could be partly because of the lower training volume of middle-distance runners. Nonetheless, exclusively concentric LV hypertrophy has been reported in professional middle-distance runners undergoing voluminous training, including the most rigorous forms of HIIT [26]. Given this ambiguity, the present study aims to determine how cardiac dimensions change in response to volume-progressive HIIT training when performed by moderately pre-conditioned young amateur athletes.

## 2. Materials and Methods

### 2.1. Subjects

Eight (six males and two females) young (mean (SD) age of 18.2 (1.0) years; range 16 to 19 years) Caucasian dinghy sailors (height of 1.75 (0.10) m; body mass index of 20.3 (1.8) kg·m^−2^) from a sailing sports school with 3–4 years of training history in that sport were involved in the study during their winter preparatory training period (the sailing off-season). The study was preceded by 6 weeks of easy recreational training 2–3 times a week after the end of the competitive season.

The study was conducted in alignment with the recent update of the Declaration of Helsinki and approved by the Lithuanian Sports University Biomedical Research Ethics Committee (No. TRS(M)-29772, 24 April 2014). All subjects and the parents of those subjects who were under 18 years of age at the start of the study read and signed the informed consent form.

### 2.2. Organization of the Study

A high-intensity interval training interventional study, designed by a professional athletics coach who also supervised all training sessions and conducted the running tests, was implemented to increase subjects’ aerobic power and running performance.

Three to five days before the start of the exercise intervention, subjects underwent a two-dimensional echocardiographic examination. A cycling aerobic power (VO_2_peak) test was conducted 5 days before, and 200 m and 2000 m running tests were performed 3 days before the training intervention. After the last training session, VO_2_peak test, echocardiographic examination, and running tests were conducted on days 3, 4, and 5, respectively.

### 2.3. Physiological and Performance Measurements

Peak oxygen consumption (VO_2_peak) was measured using the ramp incremental test on an electronically braked cycle ergometer (Ergometrics–800S; Ergoline Medical Measurement Systems, Bitz, Germany) at a pedaling cadence of ~70 rpm. Before the test, a 10-minute warm-up was performed using a combination of light pedaling and muscle stretching. The test was started with 3 min at 20 W and continued with 5 W load increments each 10 s until the pedaling cadence could not be maintained. Subjects breathed through a low-resistance mouthpiece, and oxygen consumption and carbon dioxide production were measured breath-by-breath using a wireless portable gas analyzer (Oxycon Mobile; Jaeger, Hoechberg, Germany). Before each test session, the gas analyzer was calibrated using standard gas mixtures provided by the manufacturer. VO_2_peak was measured as the highest oxygen consumption rate over consecutive 15 s periods of the test and was expressed per body mass kg. For sufficiency of the efforts used during the test, at least two of the following three criteria had to be met: VO_2_ plateau, maximal heart rate > 90% of the predicted for age (220—actual age in years), and respiratory exchange ratio > 1.1 by the end of exercise [27].

Heart rate (HR) was recorded using a Polar monitor (model T31-Coded, Kempele, Finland). Before each VO_2_peak test, the body weight of the subjects was measured using electronic scales (Tanita TBF-300, Tokyo, Japan).

Running tests were performed on the same 200 m athletics indoor track as the training program. After a supervised ~20 min warm-up consisting of jogging, stretching, and some specific drills, subjects individually performed an all-out 200 m run from a standing position. After 10–15 minutes of rest, subjects completed a self-paced 2000 m in groups of 4–6 subjects. Subjects were instructed to cover each of the distances as quickly as possible. Time was recorded manually with a stopwatch. The air temperature during the testing and training was ~18 °C. Subjects performed all the tests and training sessions in light running shoes, shorts, and T-shirts.

### 2.4. Training Intervention

As not only the exercise intensity but also the total duration of training is important for the adaptation of the cardiovascular system [21,28], in the present study, HIIT training was constructed to accumulate a substantial amount of high-intensity running over 7 weeks of intervention and not combine it with other forms of training, such as continuous workouts. At the same time, care was taken that subjects did not develop overtraining; thus, only three training sessions per week were prescribed, and the selected intervals were interchanged between and within sessions to reduce monotony (Table 1). Training of comparable volume, frequency, and duration has been shown to effectively increase aerobic power [28,29,30,31].

All subjects followed the same training program, where each workout comprised 200–1000 m intervals that increased progressively in the number of repetitions each week. Total workout time increased gradually from ~42 min per session in week 1 to ~90 min by the end of the program (range, 36 and 106 min); high-intensity exercise time increased from 12 min per session in week 1 to 30 min per session by week 7 on average. Running speed was calculated for each subject from his/her average running velocity of the pre-training 2000 m time trial, and was 95, 105, 115, and 145% for 1000 m, 600 m, 400 m, and 200 m intervals, respectively. The distance covered (but largely not the pace) during the session increased as the program progressed. Sitting and slow walking were used between intervals for recovery. Training was conducted on the 200 m synthetic indoor athletics track.

### 2.5. Echocardiographic Examination

Two-dimensional (2D) echocardiography was performed using a GE Vivid 7 system (GE Vingmed Ultrasound AS N-3190, Horten, Norway). The same experienced cardiologist performed echocardiographic recordings and measurements, being blinded to the training status of the subjects (i.e., unaware of what type of training had been implemented by the subjects before the first and between the first and second examinations). Digital loops were stored and analyzed offline (EchoPac V.6.0.0; GE Vingmed, Horten, Norway). The average of at least 3 cardiac cycles of all parameters was calculated.

Anatomic and Doppler examinations and measurements were performed according to American Society of Echocardiography recommendations [32]. Left atrial and LV end-diastolic dimensions, as well as posterior wall and interventricular septum thickness, were measured from parasternal LV long-axis images. Relative wall thickness was calculated by dividing the sum of the thicknesses of the interventricular septum and LV posterior wall by LV end-diastolic diameter. LV mass was calculated using the area–length method. The morphological LV parameters were corrected for body surface area [33]. LV ejection fraction (EF) was calculated according to Simpson’s equation. The peak early (E) and peak late (A) transmitral flow velocities, as well as early transmitral flow (E) and early diastolic mitral annular velocities at the lateral wall (E’), were measured with Doppler in the apical 4-chamber view to provide an estimate of LV diastolic function. The ratio (E/A and E/E’) was calculated. Right ventricle and right atrium dimensions were measured from apical 4-chamber views.

### 2.6. Statistics

Data are presented as means and standard deviation (SD). To test whether the changes in measured parameters occurred with training, a paired *t*-test was used. The level of significance was set at *p* < 0.05. Statistical analysis was carried out using SPSS v.21.0 (IBM Corp., Armonk, NY, USA).

## 3. Results

The subjects were carefully supervised during the training intervention and completed at least 95 percent (20 of 21) of the prescribed training sessions. During intervals, HR increased to ~190 bpm on average and recovered to ~130 bpm by the next interval. In response to 7 weeks of conditioning with HIIT, there was an improvement in running performance in sprint (200 m) and 2000 m distances, and there was also a moderate increase in VO_2_peak (from 44.1 (7.2) to 46.2 (8.3) mL·kg^−1^·min^−1^, *p* = 0.02). The HRpeak did not change significantly (*p* = 0.95), while resting HR tended to decrease (*p* = 0.05, Table 2).

**Figure 1:**
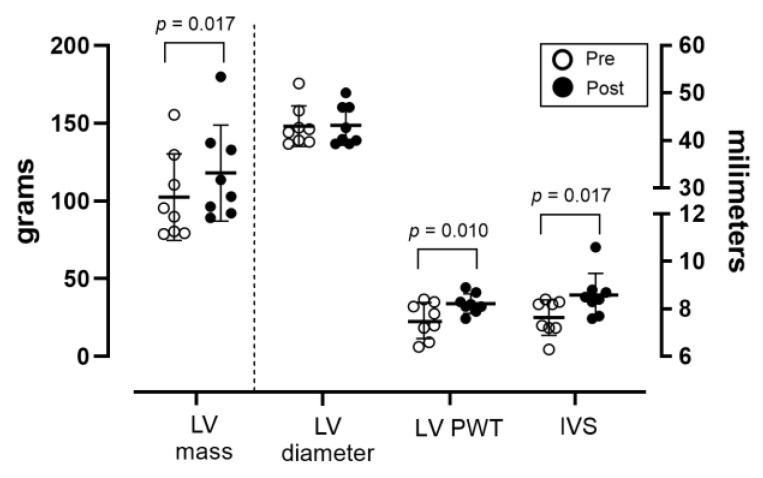
Changes in left-ventricular (LV) mass, LV internal diameter, posterior wall thickness (PWT), and intraventricular septum thickness (IVS) in response to training (pre vs. post).

There was an increase in the LV mass due to the thickening of the posterior wall and septum, but not enlargement of the LV chamber internal diameter (Figure 1). There was a small increase in left atrium diameter (*p* < 0.01) but not in other cardiac chambers, and LV diastolic function as estimated by E/A and E/E’ ratios did not change (Table 2). The ejection fraction did not change significantly, and it was above 55 percent in all of the subjects before and by the end of the training intervention.

## 4. Discussion

The results of this study show that structural changes in the hearts of young amateur athletes in response to high-intensity interval training (HIIT) of progressively increased volume are mostly due to thickening of LV walls and not chamber dilation. Despite performing just over 1 h of intense exercise per week, a slight increase in left atrium diameter was also detected. To the best of our knowledge, this is, so far, the only longitudinal study on cardiac remodeling in response to solely an HIIT program in athletes.

### 4.1. Factors of Cardiac Remodeling in Response to Exercise Training

One of the most effective modes of structural cardiac remodeling in athletes is dynamic endurance exercise such as running [34]. Total training volume is directly related to LV chamber size and LV mass in both adult and adolescent athletes [35] and soldiers [8]. The results of the current study highlight that quite substantial changes in cardiac structure (increase in LV wall thickness of ~11% and LV mass of ~24%) are possible over 7 weeks of training with as little as ~75 min of intense training per week, and imply that intensity of endurance training is critical for cardiac adaptation and may compensate for the lower training volume, as has been demonstrated in a rat model [36]. From another perspective, HIIT could be a trigger for a different type of structural cardiac remodeling as opposed to continuous training mode. The type of structural cardiac adaptation in our study is the same as in professional middle-distance runners undergoing voluminous training, including severe forms of HIIT and possessing exclusively concentric LV hypertrophy [26]. Increased myocardial mass in response to HIIT could be regarded as physiological adaptation (as also evidenced by unchanged LV function, even though measured at rest) associated with the improved performance, as shown in a previous study of distance runners [11].

It has long been a general agreement that exercise training of at least 3 h per week is required to elicit morphological cardiac adaptations [37,38]. However, later, it has been established that 3 h per week of leisure time physical activity could be enough to induce detectable cardiac hypertrophy and dilation in healthy adults [39]. The data on the required minimal activity level to induce cardiac adaptation remain controversial and may depend on multiple factors such as volume, duration, type, and intensity of exercise, as well as the gender of the participants and exercise mode. In previously sedentary women, 11 weeks of progressive continuous jogging for up to 3–3.5 h (about 30 km) per week in preparation for a 10 km race increased aerobic power (VO_2_max) but not cardiac morphological changes [40]. In similar studies on previously untrained individuals, 24 weeks of progressive endurance training up to ~3 h per week in preparation for a 12 km race increased VO_2_max, LV chamber size, and wall thickness in men [2], and 17 weeks of running up to ~3 h per week in preparation for the first marathon increased LV chamber size and wall thickness but not VO_2_max in both genders [41]. In another study of previously untrained individuals preparing for a marathon race, progressive running training for 12 months with up to ~8 weekly hours of exercise increased VO_2_max and induced balanced LV remodeling with LV dilation and wall thickening, with the former occurring with some lag [42]. Likewise, in previously untrained men, intense progressive endurance running plus cycling training of 3–3.5 h per week substantially increased VO_2_max and balanced LV hypertrophy within 7 weeks [43].

As adaptation to exercise training depends on the training background and fitness level of the subjects, the results of the present study on amateur athletes cannot be directly extrapolated to either sedentary individuals or well-trained athletes. Like other types of training, HIIT could be performed at a vast range of intensities, durations, and frequencies, and athletes usually combine it with moderate-intensity continuous training on other days [23] as well as resistance exercises, which in real-life situations end with a continuum of individually tailored training programs, making longitudinal studies of the effects of HIIT in high-profile athletes practically impossible.

### 4.2. Plausible Mechanisms of HIIT-Induced Cardiac Adaptation

As predicted and aimed for, the HIIT intervention improved both sprint and endurance running abilities and increased cycling VO_2_peak. As HRpeak did not change with the training, besides peripheral (muscular) adaptations, it could be that LV wall thickening rendered increased stroke volume (SV) and cardiac output [42] as a result of more powerful systolic contraction, and, thus better oxygen delivery to the active muscles, as proposed earlier [12]. According to the law of Laplace, an increased myocardial wall thickness with unchanged chamber volume reduces tension within the wall, rendering reduced energy demands and oxygen consumption per unit of muscle mass, thus contributing to improved fatigue resistance and ability to maintain SV and cardiac output during prolonged exercise, leading to increased working capacity. In addition to enhanced myocardial contractility, the increased maximal SV could also be mediated via improved LV relaxation capacity during exercise [44]. However, if judged from unchanged LV chamber diameter, SV could be implied to increase negligibly at best, thus probably only a small increase in VO_2_peak was evident, and even that could be ascribed largely to peripheral adaptations [45]. It is possible that increased systemic blood pressure during HIIT sessions triggered hypertrophy of the myocytes [46]. In support, LV wall thickness of endurance athletes has been shown to correlate with blood pressure immediately after intense exercise [47].

As one of the prerequisites of LV chamber dilation is considered to be a large SV during exercise, and as SV in endurance athletes is more closely related to exercise intensity compared with untrained individuals [44,48,49,50], a tentative explanation for LV chamber dilation in our study may be the HIIT performed in sufficiently large volume. In support, a much larger SV is evoked with 30 s all-out sprinting than a more endurance-type continuous incremental test to exhaustion [51]. Interestingly, the mode of the recovery (active or passive) between the high-intensity exercise bouts does not seem to affect SV between exercise bouts, at least in well-trained cyclists [52].

HIIT has been shown to acutely increase serum testosterone levels in adolescent endurance athletes [53] and untrained subjects [54] and could stimulate cardiac hypertrophy. Indeed, increased myocardial mass in steroid-abusing bodybuilders compared with non-users [55] as well as increased serum testosterone and its correlation with LV mass (r = 0.50, *p* < 0.001) in male endurance runners [4] have been found. However, voluminous endurance training is associated with a substantial reduction in resting serum testosterone [56] and increased LV wall thickness and mass [12].

Exercise training mediated an increase in blood volume [19,57], in combination with increased diastolic filling times (lowered HR) during rest and submaximal exercise, could favor eccentric LV remodeling. However, while training intensification with the addition of HIIT for a total of approximately 1 h per week for 3 weeks increased VO2max in well-trained athletes, it was in sufficient to alter plasma or blood volume [58]. On the other hand, HIIT increased left atrium volume in the current study and another study [23], an adaptation seen also in elite females performing high-volume repeated sprint training [17], and left atrium size has been shown to correlate with VO_2_peak [59].

During running (unlike cycling, rowing, swimming, etc.), the shocks generated by feet striking the surface meet and sum up at the arterial walls with pressure waves produced by each LV systole to generate pronounced fluctuations in pulse pressure (PP) and thus continuously changing cardiac load [60]. The extent to which the beat phenomenon manifests depends on the strength and frequency of both the foot contacts to the ground and the cardiac contractions. During HIIT sessions, step rate and HR tend to be similar in most young athletes, producing a more uniform pattern of PP (lengthier waves, i.e., lower oscillation in PP), which could be a factor of different cardiovascular adaptation to HIIT compared with less intense running training, where step rate substantially exceeds HR producing frequent PP waves. While average PP in runners during intense exercise has been shown to correlate with LV hypertrophy [61], the importance of individual PP wave patterns remains to be disclosed.

### 4.3. Limitations

The participants of the present study were young amateur athletes of both genders, and the overall sample size was still small, even if a similar number of subjects participated in other HIIT interventional studies [22,62]. While the LV wall thickness increased similarly between the genders (note only two females in the sample), these finding merits corroboration in larger-scale studies with larger sample sizes of male and, especially, female athletes. We used cycle ergometry rather than treadmill running to estimate the change in aerobic capacity. Neither running nor cycling was a specific activity for our subjects; thus, it could be expected that aerobic capacity also increased during running mode, which remains to be tested. Sailing is considered a technical sport of low dynamic and high static components as well as of low overall impact to increase heart size [63,64]. However, endurance-type cross-training or off-season conditioning, such as HIIT implemented in the current study, may add benefits for cardiovascular adaptations important for endurance exercise capacity, specifically required in Olympic sailing, the races of which extend for prolonged times.

A relatively short time of the HIIT intervention in our study makes it difficult to definitely conclude that such type of training would not result in different cardiac adaptation if performed for longer and/or in different populations [65]. For instance, while one study has found that intensified runners’ training with more than doubling training hours for 3 months resulted in LV dilation without wall thickening [66], another one detected quite the opposite (concentric LV remodeling) with increased running volume for 12 months [12]. An increased chamber volume and thinning of the LV wall during the progress of a professional road cycling career [13] and highly increased LV chamber diameter in ultramarathon runners [67] have also been reported. While our subjects were meticulously supervised in all training sessions during the 7 weeks of intervention and had nearly 100 percent of session attendance, such intense training is most likely too demanding to be continued for more extensive periods in this young amateur athletic population, but could be feasible in a sample of more professional athletes.

## 5. Conclusions

This study shows that as little as 8.5 h of high-intensity interval running training, comprising in total ~127 km of running, accumulated progressively over 7 weeks (i.e., just over 1 h or ~18 km per week on average) was sufficient to improve running performance, increase aerobic power, and induce left-atrial dilation and left-ventricular wall thickening, but not ventricular chamber enlargement, in young amateur athletes.

## Figures and Tables

**Table 1:** Training program used during the intervention period.

Week No.	Session No.	Intervals (m)	No. of Intervals per Session	Rest Between Intervals (min)	Total Session Time (min) *	Total Distance per Session (m)	Total Distance per Week (m)	HIE Time per Session (min)	HIE Time per Week (min)
0	1	2000 + 200 (both all-out)	2	10–15	11	2200		9	
1	2	400	6	3	37	2400	9400	9	37
	3	200	10	2	36	2000		6	
	4	1000	5	4	52	5000		22	
2	5	5 × (200 + 1000) ^#^	10	2; 4 ^&^	68	6000	17,800	28	75
	6	8 × (200 + 400) ^#^	16	2; 3 ^&^	67	4800		17	
	7	5 × (400 + 1000) ^#^	10	3; 4 ^&^	75	7000		30	
3	8	200	14	2	46	2800	13,800	8	55
	9	400	10	3	55	4000		15	
	10	1000	7	4	70	7000		32	
4	11	10 × (400 + 200) ^#^	20	2; 3 ^&^	82	6000	21,600	22	88
	12	6 × (1000 + 200) ^#^	12	2; 4 ^&^	76	7200		30	
	13	6 × (1000 + 400) ^#^	12	3; 4 ^&^	88	8400		36	
5	14	400	12	3	64	4800	19,200	18	80
	15	10 × 600 + 2 × 200	12	3	73	6400		26	
	16	1000	8	4	78	8000		36	
6	17	10 × (400 + 200) ^#^	20	3	90	6000	23,200	20	89
	18	8 × (1000 + 400) ^#^	16	3	105	11,200		22	
	20	400	15	3	77	6000		47	
7	21	8 × (1000 + 200) ^#^	16	3; 4 ^&^	106	9600	19,600	40	84
	22	1000	10	3	84	10,000		44	
8	23	2000 + 200 (both all-out)	2	10–15	10	2200		8	
			**Total**		**1440**	**126,800**		**525**	

HIE, high intensity exercise; * total time of high intensity intervals and recovery periods in between; time for warm-up and cool-down not included (these were jogging and dynamic stretching drill and were never longer than 10 min per both warm-up and cool-down per training session); ^#^ intervals were mixed, e.g., 200 m–400 m–200 m–400 m, and on; ^&^ the first number represents break after the shorter interval, and the second number represents that after the longer interval.

**Table 2:** Echocardiographic indices before and after 7 weeks of HIIT. Data are mean (SD).

	Pre-Training	Post-Training	*p* Value
Body weight, kg	62.9 (11.3)	63.1 (11.3)	0.45
Heart rate, bpm	68.4 (9.5)	64.3 (8.9)	0.05
LV mass index, g·m^−2^	49.2 (8.0)	58.7 (6.6)	**0.02**
Relative wall thickness	0.354 (0.033)	0.387 (0.024)	0.18
Left atrium diameter, mm	32.0 (2.5)	33.5 (2.3)	**0.01**
RV diameter, mm	29.6 (2.8)	29.2 (2.1)	0.65
Right atrium diameter, mm	38.7 (2.1)	37.3 (3.7)	0.68
E/A	1.91 (0.25)	1.86 (0.34)	0.87
E’, cm·s^−2^	17.4 (3.6)	17.0 (1.3)	0.69
E/E’	5.35 (0.92)	5.35 (0.94)	0.70

LV, left ventricular; RV, right ventricular; E, early diastolic peak filling velocity; A, late diastolic peak filling velocity; E’, peak early diastolic mitral annular velocity. Significant *p*-values are highlighted in bold.

## Data Availability

The original contributions presented in this study are included in the article. Further inquiries can be directed to the corresponding author.
